# Real-Time Monitoring of Cancer Cells in Live Mouse Bone Marrow

**DOI:** 10.3389/fimmu.2018.01681

**Published:** 2018-08-02

**Authors:** Sung Hwan Lee, Sang A Park, Yunyun Zou, Sang-Uk Seo, Chang-Duk Jun, Woo Jung Lee, Young-Min Hyun, Nam Hoon Cho

**Affiliations:** ^1^Department of Surgery, Yonsei University College of Medicine, Seoul, South Korea; ^2^Department of Anatomy, Yonsei University College of Medicine, Seoul, South Korea; ^3^Brain Korea 21 Plus Project for Medical Science, Yonsei University College of Medicine, Seoul, South Korea; ^4^Department of Biomedical Sciences, Wide River Institute of Immunology, Seoul National University College of Medicine, Seoul, South Korea; ^5^School of Life Sciences, GIST, Gwangju, South Korea; ^6^Immune Synapse and Cell Therapy Research Center, GIST, Gwangju, South Korea; ^7^Department of Pathology, Yonsei University College of Medicine, Seoul, South Korea

**Keywords:** two-photon microscopy, intravital imaging, bone marrow microenvironment, tumor cell dormancy, cancer cell

## Abstract

Disseminated tumor cells in the bone marrow environment are the main cause of systemic metastasis after curative treatment for major solid tumors. However, the detailed biological processes of tumor biology in bone marrow have not been well defined in a real-time manner, because of a lack of a proper *in vivo* experimental model thereof. In this study, we established intravital imaging models of the bone marrow environment to enable real-time observation of cancer cells in the bone marrow. Using these novel imaging models of intact bone marrow and transplanted bone marrow of mice, respectively, *via* two-photon microscopy, we could first successfully track and analyze both the distribution and the phenotype of cancer cells in bone marrow of live mouse. Therefore, these novel *in vivo* imaging models for the bone marrow would provide a valuable tool to identify the biologic processes of cancer cells in a real-time manner in a live animal model.

## Introduction

Early systemic metastasis is a major feature of major cancers even after margin-negative resection of a primary cancer lesion (1, 2). Circulating tumor cells derived from primary cancer lesion can be disseminated to secondary organs including bone marrow blood, lymph node, and distant organ *via* blood vessels or lymphatic channels and can cause early systemic recurrence regardless of definite treatment (3, 4). The majority of cancer-related deaths are due to the involvement of metastatic tumors originating from disseminated cancer cells (5, 6). Contrary to other malignancies showing a dormant tumor phenotype in secondary organs, most notably in breast cancer, it has been known that aggressive cancer types such as pancreatic cancer can overcome easily or bypass suppressive microenvironment compared to other malignancies having a relatively long dormant period (3, 7–9). Therefore, the mechanism of an awakening or activation of cancer cells in secondary organs should be investigated in order to prevent early systemic metastasis even in resectable cancer. The tumor biology of cancer cells in the bone marrow environment, the main site of minimal residual disease after curative cancer treatment, should be investigated in order to identify the specific process of systemic metastasis in most solid cancers (3, 10, 11). However, the lack of an *in vivo* experimental model for cancer cells in the bone marrow environment has been a major barrier to exploring the initial process of systemic metastasis (4, 12, 13). The aim of this study is to establish intravital imaging model of cancer cells in the bone marrow environment.

## Materials and Methods

### Cancer Cell Lines and Culture Conditions

Human cancer cell lines MCF7 and AsPC-1 were obtained from the American Tissue Culture Collection (ATCC) and grown in RPMI-1640 medium (ThermoFisher, Waltham, MA, USA) supplemented with 10% fetal bovine serum, 100 U/ml penicillin, and 100 µg/ml streptomycin. These cell lines were authenticated by standard short tandem repeat DNA typing methodology before purchase from the ATCC. Mouse cancer cell lines 4T1, MMT060562, SL4, Lewis Lung Carcinoma, and Pan02 were provided by Anticancer Inc. (San Diego, CA, USA). All cell lines were maintained and incubated in a humidified CO_2_ chamber maintaining 37°C and 5% CO_2_.

### Establishment of Fluorescent Cancer Cell Lines

Cancer cell lines were tagged with green or red fluorescent protein (GFP or RFP) using a lentiviral transduction system. GFP and RFP were cloned into pLenti CMV/TO Puro empty vectors purchased from Addgene (Cambridge, MA, USA). Lentiviruses were prepared by cotransfection of packaging vectors pMD2G, pMDLg/pRRE, and pRSV-Rev, and pLenti CMV/TO Puro-GFP or RFP into HEK 293T cells. GFP or RFP cancer cell lines were established by lentiviral infection using transduction technique, and selection was performed for 1–2 weeks using 1 µg/ml of puromycin.

### Proliferation Assay for Cancer Cells

Assays for the proliferation of cancer cells in the bone marrow environment were performed by measuring fluorescence intensity every day. Fluorescence intensity was measured using a fluorescence microplate reader (Varioskan Flash Multimode Reader, Thermo Scientific, USA) using bottom optic readings. Excitation/emission wavelengths were 488/507 nm for GFP and 553/574 nm for RFP.

### Western Blot Analysis

Cells were trypsinized and lysed with a Pro-Prep protein extraction kit (iNtRON Biotechnology, Seongnam, Korea). Equal amounts of protein extracts (20 µg) were separated by sodium dodecyl sulfate-polyacrylamide gel electrophoresis and transferred to a nitrocellulose membrane (Invitrogen). Blots were blocked with 5% nonfat dry milk at room temperature. The blots were incubated with antibodies for ERK1/2 (sc-93, Santa Cruz Biotechnology, 1:5,000), phospho-ERK1/2 (sc-101760, Santa Cruz Biotechnology, 1:1,000), p38 (sc-7972, Santa Cruz Biotechnology, 1:1,000), and phospho-p38 (sc-7973, Santa Cruz Biotechnology, 1:500), followed by incubation with peroxidase-labeled secondary antibodies. Immunoreactive proteins were visualized using an enhanced chemiluminescence detection kit (Santa Cruz Biotechnology). Blot images were captured using an ImageQuant LAS 4000 biomolecular imager (GE Healthcare Life Sciences). Quantification of Western blots was conducted by ImageJ (Image Processing and Analysis in Java, NIH, USA) software. After preparation of the blot images following digital scanning, defining regions of interest (ROI) and measurements for each ROI were performed consecutively.

### Animals

C57BL/6 and BALB/c nude mice were purchased from the Jackson Laboratory (ME, USA) *via* Orient Bio (Sungnam, Korea). CX3CR1-GFP (14) and LysM-GFP (15) transgenic mice were obtained, and genotyping for each strain was performed according to the corresponding reference. All mice were maintained in a pathogen-free environment in the animal facility at Avison Biomedical Research Center in Yonsei University College of Medicine, and the animal experiments were approved by the Institutional Animal Care and Use Committees at the Yonsei University College of Medicine.

### Mouse Imaging Models for Two-Photon Intravital Imaging

To observe the bone marrow environment effectively, we established two imaging window models, a calvarial window in the skull and a dorsally transplanted femur bone marrow window. A natural imaging window for calvarial bone marrow in skull bone had been previously established. Meanwhile, however, the dorsally transplanted femur bone marrow model was newly created to investigate the exact biology of cancer cells in bone marrow. Whole surgical procedures were performed carefully by a single hepatobiliary surgeon who has abundant experience in microsurgery. The operative procedures for each intravital imaging model were performed as described in the following sections.

### Establishment of the Imaging Window for Calvarial Bone Marrow

After anesthesia of fluorescence expressing mice (CXCR1-GFP or LysM-GFP mouse) using Zoletil^®^ (Virbac Korea, Seoul, Korea) *via* intraperitoneal injection, mice was placed onto a stereotactic heating plate (Live Cell Instrument, Seoul, Korea) to maintain body temperature. The scalp was removed with scissors with a 1.5-cm radius (Figure S1A in Supplementary Material), and then, acrylic resin was applied around the exposed skull area (Figure S1B in Supplementary Material). The fixation ring was attached to the pre-applied acrylic resin and then assembled with a stereotactic head fixation device (Live Cell Instrument, Seoul, Korea) attached to the heating plate (Figure S1C in Supplementary Material).

### Preparation of Donor Mouse and Processing of Femur Bone Graft

**Figure 1:**
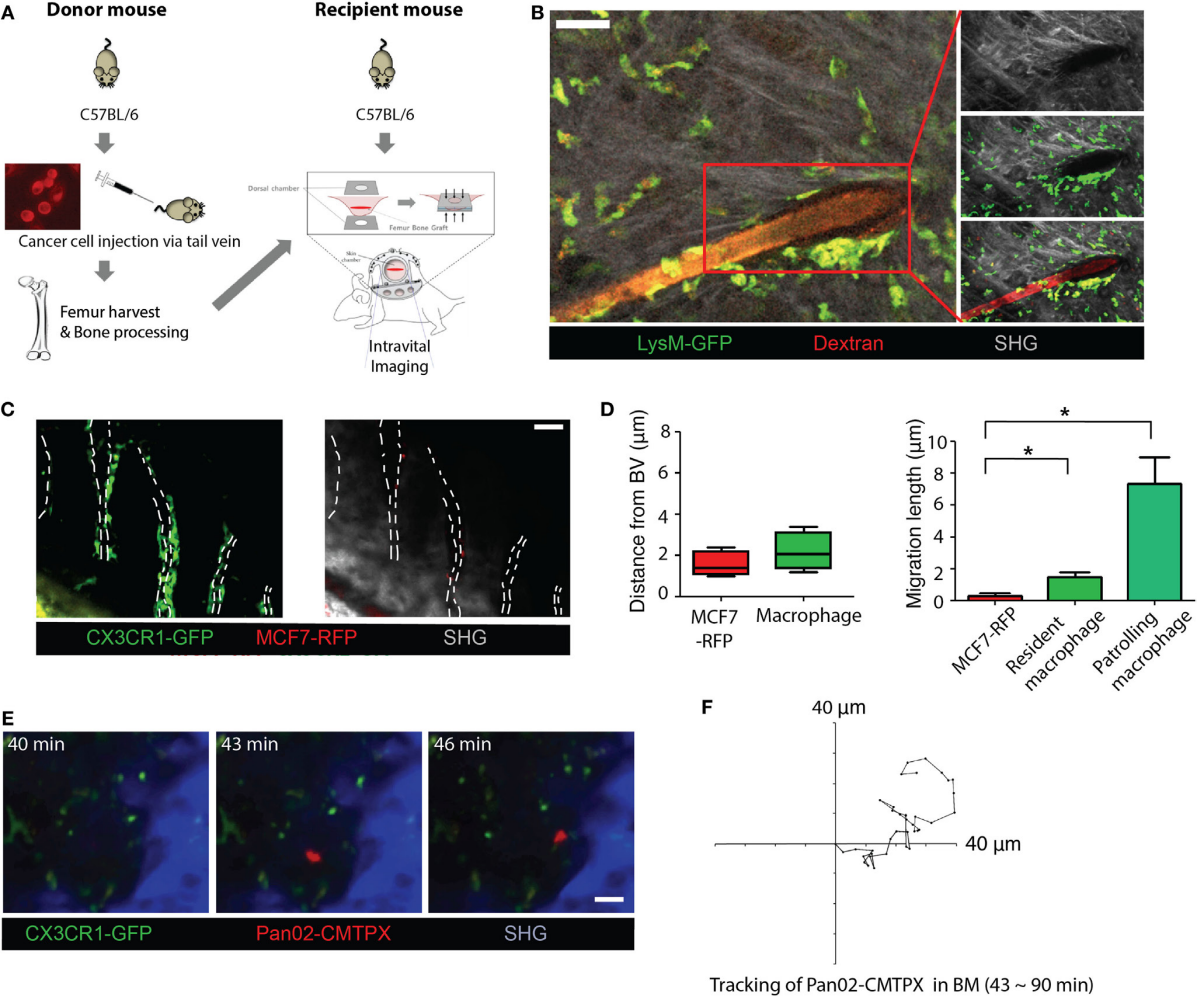
Establishment of a dorsally transplanted bone marrow model for intravital imaging and application of intravital imaging for cancer cells in the bone marrow environment. **(A)** Schematic presentation of the dorsally transplanted femur bone marrow imaging model using donor and recipient mice (detailed procedures for the formation of imaging window and the preparation for intravital imaging were described in Section “Materials and Methods”). **(B)** Cell viability of dorsally transplanted femur bone marrow was confirmed *via* 4D live imaging tracking, and vascular connections between the donor bone marrow and recipient fascia layer were identified by 3D structural analysis (scale bar = 30 µm and see Videos S1 and S2 in Supplementary Material). **(C)** GFP-expressing monocytes and macrophages in bone marrow and RFP expressing cancer cells (MCF7-RFP) (dotted line: blood vessels, scale bar = 50 µm and see Video S3 in Supplementary Material). **(D)** Cancer cells (MCF7-RFP) were located at perivascular areas and distances of cancer cell migration in bone marrow were significantly lower than those of macrophages in the Mann–Whitney *U* test (migration length; MCF7-RFP vs. Resident macrophage = 0.21 ± 0.18 vs. 1.24 ± 0.67 µm, MCF7-RFP vs. patrolling macrophage = 0.21 ± 0.18 vs. 7.21 ± 2.14 µm, *p < 0.05). Data were averaged from independently repeated experiments three times. **(E)** Rarely observed active cancer cells in the bone marrow environment (scale bar = 30 µm and see Videos S4 and S5 in Supplementary Material). Pan02-CMPTX; red-labeled Pan02 cells. **(F)** Serial tracking of an active cancer cell (Pan02-CMPTX) in the bone marrow environment.

After harvesting cultured cancer cells *in vitro* using a 100-mm dish, 1-ml syringes with a 31-G needle were prepared with 0.5 ml of injectable normal saline mixed with a predefined number of cancer cells. The cancer cells were injected into the tail vein of a donor mouse (fluorescence expressing mouse: CX3CR1-GFP or LysM-GFP mouse) for femur bone transplantation with a prefilled 1-ml syringe without anesthesia. After a period of mouse breeding in an animal facility for mice over 1–7 days after the injection, the femur bone of the donor mouse was harvested carefully after euthanasia in a CO_2_ chamber (Figure 1A). The extracted femur bone of the donor mouse was immediately processed for long bone transplantation. First, both epiphyses of the femur bone were excised using a surgical scalpel blade (No.10) and one side of the femur bone cortex was removed with fine micro-dissecting spring scissors (JD-S-16, Jeung Do Bio and Plant Co., Ltd., Seoul, Korea) longitudinally. Approximately 40% of the circumference of the femur bone shaft was removed carefully. To minimize the iatrogenic damage of exposed sections of femur bone marrow, the procedure of bone opening was carefully performed under a magnifying glass (5× magnification, 100 mm diameter convex lens) with moisturizing with phosphate-buffered saline (PBS) (GIBCO, Thermo Fisher Scientific, USA) (Figures S2A,B in Supplementary Material).

### Establishment of the Imaging Window for Transplanted Femur Bone Marrow in Recipient Mice

During the femur bone preparation in the donor mouse, formation of the imaging window for the dorsal chamber in recipient mice (C57BL/6 or BALB/c nude mice) was simultaneously carried out in keeping with the progress of femur bone harvesting and processing in donor mouse. Recipient mice were anesthetized by Zoletil^®^ injection into the intraperitoneal cavity and placed on a heating plate to maintain body temperature. For stable installation of a dorsal chamber kit (SM100, 27 mm titanium, APJ trading, USA), the back skin was sutured and retracted in an upward direction. The unilateral back skin with round shape was removed by fine micro-dissecting spring scissors along the position for the microscope cover glass (GL100, 12 mm, 0.13 mm thick, APJ trading, USA) for intravital imaging (Figure S2C in Supplementary Material). The dorsal chamber kit was assembled into the retracted back skin of recipient mice, except for cover glass equipment. Immediately after completing femur bone processing of the donor mouse, the exposed bone marrow side of the femur graft was placed onto the back skin of a recipient mouse after being excised in a round shape for cover glass positioning. After delicate dropping of PBS around the femur bone graft, the cover glass was placed and fixed to the dorsal chamber (Figures S2B,C in Supplementary Material).

### Two-Photon Intravital Microscopy

Mice were anesthetized using *via* intraperitoneal injection of Zoletil at a dose of 30 mg/kg during imaging procedures. Long-term anesthesia was performed using the inhalation agent isoflurane. A staging system (manual XY stage and microscope mounting plate) was set up using Live Cell Instrument Korea (Seoul, Korea), and two-photon microscopy (LSM7MP, Carl-Zeiss, Germany) was used for imaging data generation. Zen software (Carl-Zeiss) was used for image acquisition and basic image analysis. For two-photon excitation, light of 880–900 nm wavelength was used for imaging green, red, and second harmonic generation. Images were acquired at a resolution of 512 × 512 pixels using step sizes of 1 µm to a depth of 30–50 µm every 30–60 s.

### Imaging Data Analysis

Fiji/ImageJ software was used for image analysis and basic image processing. IMARIS version 7 (Bitplane, USA) and Volocity software (PerkinElmer, USA) were used for 3D and 4D imaging data analysis.

### Flow Cytometry Analysis

Chronological flow cytometry analysis at baseline (control) and 1 and 7 days after cancer cell injection *via* the tail vein. Injectable saline without cancer cells was injected to control mice. The acquisition of bone marrow for flow cytometry was performed by aspiration from bone marrow of the femur bone at the day of injection (control) and 1 and 7 days after injection *via* the tail vein. Then, mouse bone marrow was obtained 1 and 7 days after cancer cell injection. Harvested bone marrow cells were washed with PBS and fixed with 4% paraformaldehyde for 10 min. The fixed cells were chilled on ice and permeabilized with 90% methanol for 30 min. Then, 1 × 10^6^ cells per experimental condition were aliquoted, washed, and resuspended in 100-µl solutions of fluorochrome-conjugated primary antibodies against DAPI (564907, BD Biosciences, USA), MHC class II (550750, BD Biosciences, USA), and CD11b (553311, BD Biosciences, USA) at the manufacturer’s recommended concentrations, and incubated for 1 h. For isotype control, fluorochrome-conjugated rabbit IgG was used at the same concentration. Cells were washed, resuspended in PBS, sorted by fluorescence, and analyzed using a FACSAria cell sorter.

### Statistical Analysis

Graphical data generation and basic statistical analyses were performed using GraphPad Prism 6 software. Comparisons between the separate groups were conducted using the Mann–Whitney *U* test for non-parametric test of continuous variables to calculate statistical probability of non-parametric test. *p*-Values less than 0.05 were considered statistically significant.

## Results and Discussion

To monitor the entire biological process of the cancer cells in the bone marrow in real-time manner, we established two different intravital imaging models to track cancer cells in the bone marrow environment of live mice by employing specific windows for the calvarium and transplanted femur, respectively. An imaging window for indigenous calvarial bone marrow in the mouse skull (Figure S1 in Supplementary Material) was adopted with a stable fixed-staging system (16). In addition, a novel imaging window to observe bone marrow in a transplanted femur was contrived using a dorsal chamber (17) (Figure 1A). To investigate whether our newly designed femur bone transplantation model works for engraftment, we transplanted MCF7-RFP cells injected bone to healthy LysM-GFP mice. Using two-photon intravital microscopy of the MCF7-RFP-carrying bone marrow transplanted LysM-GFP mice, we confirmed that the transplanted femur bone marrow was successfully engrafted as a viable live tissue after 7 days from the implantation, and intact vascular connections with a vascular network system were formed at the fascia layer of the recipient mouse (Figure 1B; Videos S1 and S2 in Supplementary Material). The transplanted bone marrow tissue was viable at all-time points at days 1–30 after the surgery. Therefore, it was feasible to perform intravital imaging of the interaction of cancer cell with leukocytes from the implanted bone marrow in the recipient mice.

Interestingly, we found that cancer cells were mainly located in the perivascular area in the bone marrow environment after intravenous injection of cancer cell lines. A total of 5 × 10^5^ of cancer cells from human (MCF7 and AsPC-1) and mouse (4T1, MMT060562, SL4, Lewis Lung Carcinoma, and Pan02) cancer cell lines were used. From 4D image analyses (3-dimensional image analyses in time lapse manner), the migration trajectory of cancer cells was significantly shorter than those of primary cells in the bone marrow environment. The relative location of cancer cells from the blood vessel was closer to blood vessel. Migration length of cancer cells in an hour was statistically shorter than the patrolling macrophage crawling around the vessel wall and resident macrophage in the bone marrow parenchyma of CX3CR1-GFP mice (Figures 1C,D; Video S3 in Supplementary Material). We also confirmed similar localization and migration pattern of other cancer cells closer to blood vessels, when other cancer cells from human (AsPC-1) and mouse (4T1, MMT060562, SL4, Lewis Lung Carcinoma, and Pan02) cancer cell lines were used for the same experiment (data not shown). When we used red-labeled Pan02 cells (Pan02-CMPTX) to further compare migration patterns, the phenotypes of cancer cells were very quiescent compared to other bone marrow components; therefore, once cancer cells extravasated through the vasculature, they appeared to simply remain close to the outside of the vessel wall. Proliferation assays (Figures S3A–C in Supplementary Material) and *in vitro* live cell imaging (Figures S3D,E and Videos S4–S6 in Supplementary Material) revealed that the growth rate of cancer cells cocultured with bone marrow cells remarkably decreased compared to those of cancer cells alone or those cocultured with NIH-3T3 cells (mouse-derived fibroblasts). In addition, low ERK/p38 ratios and expression patterns, the well-known indicator of tumor cell dormancy, were observed consistently by western blot analysis (Figures S4A,B in Supplementary Material). These data suggest a need for further investigation as to whether cancer cells acquire a dormant phenotype in this model.

**Figure 2:**
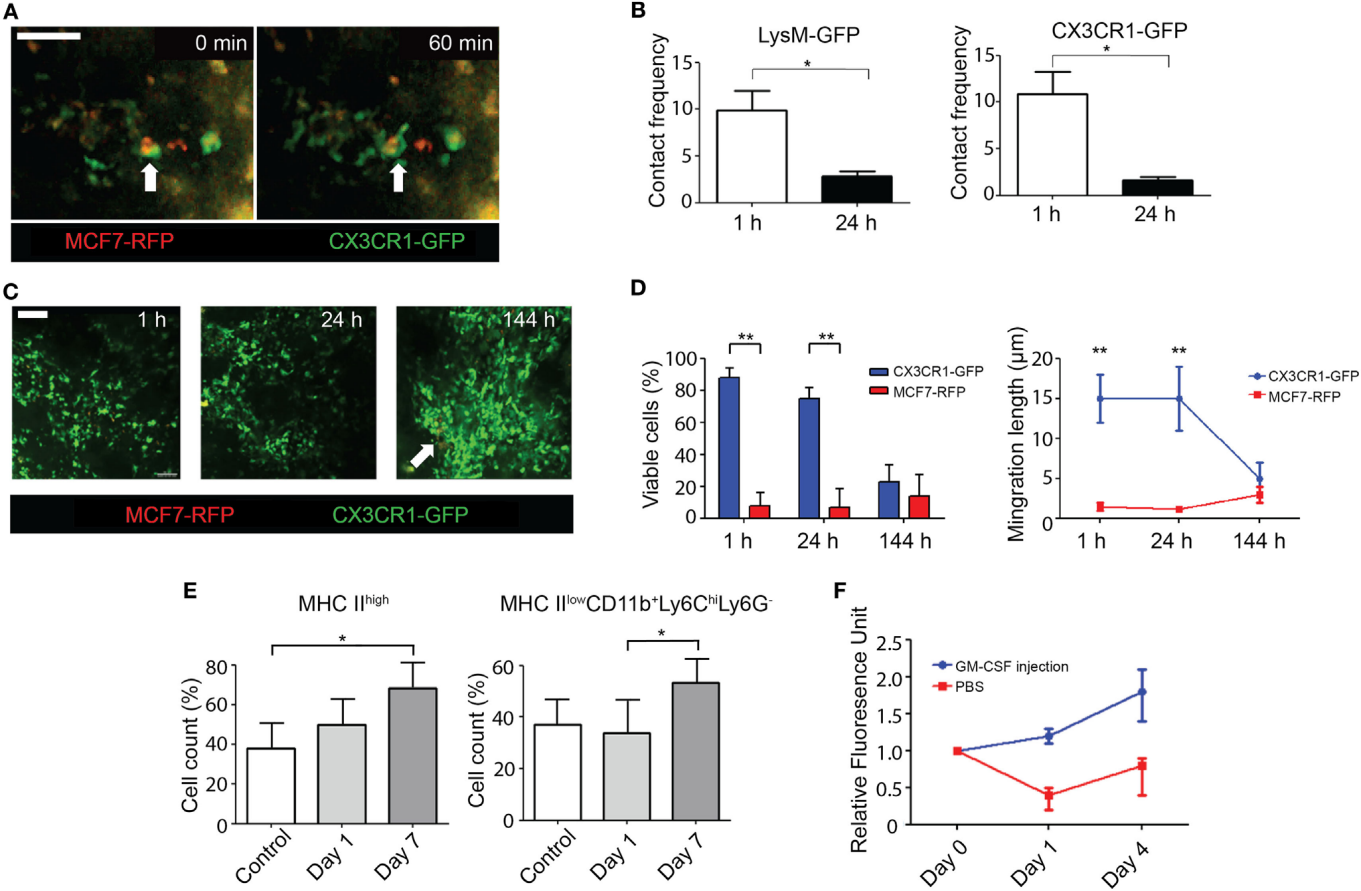
Application of intravital imaging for cancer cells in the bone marrow environment. **(A)** Engulfment of cancer cells by macrophages (white arrow) in calvarial bone marrow imaging (scale bar = 50 μm and see Video S6 in Supplementary Material). **(B)** Temporal changes in interactions between MCF7-RFP and LysM-GFP or CX3CR1-GFP cells in the calvarial bone marrow environment. Contact frequency of MCF7-RFP cells with LysM-GFP or CX3CR1-GFP cells was significantly decreased at 24 h compared to 1 h after MCF7-RFP injection *via* mouse tail vein. The Mann–Whitney *U* test was used to compare two groups. *p < 0.05. Data were averaged from independently repeated experiments three times. **(C)** Captured bone marrow images in 1, 24, and 144 h (white arrow: clustered proliferation of MCF7-RFP) after gemcitabine injection intravenously (scale bar = 50 µm and see Videos S7–S9 in Supplementary Material) in the calvarial bone marrow model. **(D)** Viable cell counts of CX3CR1-GFP and MCF7-RFP (viable cells in 1 h; 84.54 ± 7.81% of CX3CR1-GFP vs. 8.29 ± 7.64% of MCF7-RFP, 24 h; 73.24 ± 7.64% of CX3CR1-GFP vs. 7.82 ± 7.24% of MCF7-RFP, **p < 0.01) and temporal changes in migration pattern after gemcitabine injection in the calvarial bone marrow model (migration length in 1 h; 14.98 ± 2.56 µm in CX3CR1-GFP vs. 1.22 ± 0.84 µm in MCF7-RFP, 24 h; 14.92 ± 3.84 µm in CX3CR1-GRP vs. 0.94 ± 0.28 µm in MCF7-RFP, **p < 0.01). The Mann–Whitney *U* test was used to compare two groups. A representative data were shown from independently repeated experiments three times. **(E)** Chronological flow cytometry analysis showing control and 1 and 7 days after cancer cell injection *via* the tail vein in a mouse that was not used for intravital imaging. Injectable saline without cancer cells was injected to control mice. The acquisition of bone marrow for flow cytometry was performed by the aspiration from bone marrow of the femur at the day of injection (control) and 1 and 7 days after the injection *via* tail vein. MHC II^high^ cells and MHC II^low^CD11b^+^Ly6C^hi^Ly6G^−^ cells were significantly increased in day 7. The Mann–Whitney *U* test was used to compare two groups (cell count of MHC II^high^; 39.24 ± 8.84% in control vs. 67.26 ± 12.45% in day 7, MHC II^low^CD11b^+^Ly6C^hi^Ly6G^−^; 35.45 ± 8.74% in day 1 vs. 53.48 ± 17.31% in day 7, *p < 0.05). Data were averaged from independently repeated experiments three times (see Figure S5 in Supplementary Material). **(F)** Qualitative analysis of relative fluorescence of MCF7-RFP with or without GM-CSF injection before injection and 1, 4 days after injection in dorsally transplanted bone marrow model.

However, active cancer cells derived from the mouse cancer cell line Pan02 in bone marrow environment showing active movement like bone marrow stromal cells were observed very rarely and were closely tracked (Figures 1E,F; Videos S7 and S8 in Supplementary Material). Two-photon intravital imaging of cancer cells in calvarial bone marrow also verified that cancer cells were in active contact with resident macrophages in the bone marrow environment of CX3CR1-GFP mice (Figure 2A; Video S9 in Supplementary Material). Even after serially sustained contact with CX3CR1-GFP or LysM-GFP expressing cells, the cancer cells were consistently observed to be viable without undergoing immune clearance. Interaction between cancer cells and CX3CR1-GFP or LysM-GFP expressing cells were significantly decreased 24 h after i.v. injection of cancer cells in both LysM-GFP and CX3CR1-GFP mouse, compared to that within 1 h after i.v. injection of cancer cells (Figure 2B). This difference in interactions between cancer cells and stromal cells in the bone marrow may result from the fact that temporal change of local innate immunity toward cancer cells might be the reason of cancer dormancy in the bone marrow environment. Further evaluations of this finding should be done to confirm these observations. Flow cytometry analysis also revealed that the subpopulation of myeloid lineage changed over time after cancer cells entry into the bone marrow environment (Figure 2E; Figures S5A,B in Supplementary Material).

The effects of chemotherapeutic agents on dormant cancer cells in the bone marrow environment are not yet known because of the absence of a suitable experimental model. Therefore, an interventional experiment was performed using our novel model to investigate the impact of a chemotherapeutic agent on cancer cells, as well as on the bone marrow environment. After injection of the chemotherapeutic agent gemcitabine, the main drug used for first or second-line chemotherapy of several solid cancers, such as pancreatic cancer, ovarian cancer, lung cancer, and breast cancer, we observed that the cell number and movement of various bone marrow components were significantly decreased. However, the number of cancer cells remained consistent; cancer cells appeared to be less affected by the chemotherapeutic agent than other normal cells in bone marrow (Figures 2C,D; Videos S10–S12 in Supplementary Material). These initial findings from the intravital imaging model indirectly suggest that current clinical policy regarding adjuvant chemotherapy might be creating a paradoxical effect, in which chemotherapeutic agents induce the relative activation of cancer cells in suppressive microenvironments, such as cancer cells in the bone marrow environment. A confirmative study with a robust experiment design is mandatory to prove these initial findings. Another example of an interventional approach using our model of the bone marrow environment showed that the morphology and movement of immune cells and stromal cells in the bone marrow environment were significantly increased immediately after i.v. injection of granulocyte-macrophage colony factor (GM-CSF). In coculture with murine cancer cell lines, active movement and sudden disappearance of the cancer cells from bone marrow environment was observed in a small portion of the engulfed tumor cells in the perivascular area of the mouse bone marrow. Bone marrow stimulation by GM-CSF was a significant factor for reactivation of cancer cells in the mouse bone marrow environment (Figure 2F). These findings indicate that GM-CSF administration due to neutropenia after chemotherapy could unintentionally cause the activation of cancer cells in the bone marrow environment. Further investigations into the detailed mechanisms of this paradoxical phenomenon should be performed to address these concerns.

Bone marrow is a representative depot for the distribution of cancer cells clinically known as minimal residual disease (18–21). Nonetheless, only a few studies on cancer cells in the bone marrow environment have shown direct experimental evidence of their biology. In this study, we have successfully established two imaging models for intravital observation of the bone marrow in both the skull calvarium and femur. These intravital imaging models can elucidate the detailed biology of cancer cells, including their distribution and biological behavior in response to specific stimulation as well as reactivation phenomenon. Femur bone marrow transplantation from a cancer cell-injected mouse to the dorsal area of a healthy mouse was first developed to identify the biology of the cancer cells in the bone marrow using two-photon intravital microscopy. The combination of the calvarial window for observing indigenous healthy bone marrow and the dorsally transplanted cancer cell-bearing bone marrow model could capture the entire process, from the bone marrow dissemination of cancer cells to systemic metastasis, as novel experimental animal models using intravital imaging. These findings are consistent with current theories of cancer cells behavior in the bone marrow environment. Additionally, our results verified that even after constant exposure to neutrophils and macrophages, the cancer cells were robustly viable in the bone marrow environment. This phenomenon provides new insights for cancer immunology in the bone marrow environment favoring immune tolerance for cancer cells. Chemotherapeutic agents caused significant damage to *in situ* bone marrow components but did not influence the viability of cancer cells in the bone marrow environment. The presence of cancer cells in bone marrow in patients has been strongly suggested to be a risk factor for metastasis (22–24). However, clinical implications of cancer cells in bone marrow-targeting strategies for preventing metastasis could only be addressed when detailed mechanistic analysis of cancer cell biology in bone marrow was undertaken in preclinical models (4, 25, 26). It is, therefore, inevitable that an *in vivo* bone marrow cancer cell model should be developed for real-time monitoring and tracking (27).

There are several limitations in this study regarding insufficient experimental validations for specific biologic process and limited scope to perivascular niche for the cancer cells in the bone marrow environment. Although the experiments of intravital imaging were conducted as far as possible from the interface of implantation, the method of bone marrow transplantation, which can create artificial wound repair-like process, can also affect the result of experiments using the femur bone transplantation model. The experimental set-up and initial validations, however, were primary milestone in this study. Therefore, vigorous experimental validation and subsequent functional study should be followed. Taken together in this study, we showed that the specific biologic process of cancer cells in bone marrow can be elucidated in high resolution using two-photon intravital imaging. This novel method offers a very useful tool for gaining new insights into cancer cell biology in bone marrow, especially, the identification of both dormancy and reactivation of cancer cells in bone marrow *in vivo*.

## Ethics Statement

All mice were maintained in a pathogen-free environment in the animal facility at Avison Biomedical Research Center in Yonsei University College of Medicine, and the animal experiments were approved by the Institutional Animal Care and Use Committees at the Yonsei University College of Medicine.

## Author Contributions

Study conception and design; analysis and interpretation of data; drafting of manuscript: SL, Y-MH, and NC. Acquisition of data: SL, YZ, S-US, SP, and Y-MH. Technical support: S-US and C-DJ.

## Conflict of Interest Statement

The authors declare that the research was conducted in the absence of any commercial or financial relationships that could be construed as a potential conflict of interest.

## Supplementary Material

Figure S1 Calvarial bone marrow imaging model. **(A)** The scalp was removed by scissors with a 1.5-cm radius size. **(B)** Acrylic resin was applied around the exposed skull area to attach a fixation ring for the attachment with imaging mount module. **(C)** The fixation ring was attached to the pre-applied acrylic resin and then assembled with stereotactic head fixation device attached to a heating plate. **(D)** Two-photon microscopy revealed the typical bone marrow structure of the calvarium with trabecular anatomical structures in cortical bone (scale bar = 50 µm). **(E)** The number of viable cancer cells (MCF7) in calvarial bone marrow according to the number of injected cancer cells. **(F)** The number of viable cancer cells (MCF7) in calvarial bone marrow according to the period after cancer cell injection, from 1 to 28 days (the number of injected cancer cells: 1 × 10^7^).

Figure S2 Dorsally transplanted femur bone marrow imaging model. **(A)** Bone graft processing from donor femur bone. **(B)** Final visualization of dorsal window for transplanted femur bone marrow using two-photon microscopy. **(C)** Operative procedures and mounting for intravital imaging of dorsal chamber implantation into recipient mouse. Detailed operative procedures are described in Section “Materials and Methods.”

Figure S3 Human cancer cell lines adapt to bone marrow environment by dormant phenotype. **(A)** Proliferation assay for cancer cells with or without coculture to identify the inhibition effect of cancer cell proliferation by bone marrow stromal cells. Cancer cells (MIA PaCa-2, AsPC-1, MCF-7, and MDA-MB-231) alone or in coculture with NIH/3T3 (mouse fibroblast) cells or mouse bone marrow stromal cells that were aspirated from the femur bone of C57BL/6 mice. Coculture was performed on a 6-well plate (cancer vs. fibroblast or BM stromal cell = 1:10 ratio). **(B)** Relative fluorescence units on culture day 6. Proliferation of cancer cells coculture with mouse BM stromal cells compared to NIH/3T3 was significantly decreased in MIA PaCa-2 and MCF7 cell lines in the Mann–Whitney *U* test (relative fluorescence unit of MIA PaCa-2 in day 6 relative to day 1; 4.42 ± 1.18 in coculture with NIH/3T3 vs. 1.65 ± 0.52 in coculture with mouse BM stromal cells, MCF7; 4.47 ± 0.34 in coculture with NIH/3T3 vs. 1.74 ± 0.27 in coculture with mouse BM stromal cells, **p* < 0.05). Data were averaged from independently repeated experiments three times. **(C)** Microscopy images of each cell line under bright field and green fluorescence. Bar graphs represent mean ± SD, scale bar = 30 µm. **(D)** Live cell imaging and **(E)** quantitative analysis of MCF7 cells showed restricted proliferation compared to active proliferation in monoculture and coculture with NIH/3T3 cells in the Mann–Whitney *U* test. **p* < 0.05. See Videos S10–S12 in Supplementary Material. Representative data were shown from independently repeated experiments three times.

Figure S4 Western blot analysis of ERK/p-ERK/p38/p-p38 in mouse cancer cell line monocultures vs. coculture with mouse bone marrow. **(A)** Western blot to confirm of cancer dormancy at the molecular level. Various cancer cell-lines originated from a C57BL6 mouse (MMT060562, LLC, Pan02, SL4) were cocultured with mouse bone marrow stromal cells that were aspirated from the femur bone of a C57BL6 mouse. Blotting images represent the relative protein expressions of p-ERK/ERK and p-p38/p38 in cancer cell-lines. **(B)** Quantitative analysis of western blot images with or without coculture with mouse bone marrow by the Mann–Whitney *U* test. Expression ratio of phospho-ERK with phospho-p38 between monoculture and coculture with BM were significantly different in MMT060562 and SL4 cell lines. **p* < 0.05. The mean values were quantified from pooled experiments conducted using different lysates from independent samples three times.

Figure S5 Temporal changes of myeloid lineage in mouse bone marrow after cancer cell injection *via* tail vein. **(A)** Chronological flow cytometry analysis showing control and 1 and 7 days after cancer cell injection *via* the tail vein. Injectable saline without cancer cells was injected to control mice. The acquisition of bone marrow was performed by the aspiration from bone marrow of the femur bone at the day of injection (control) and 1 and 7 days after injection *via* the tail vein. **(B)** Quantitative analysis of temporal changes for myeloid derived suppressive factors in myeloid lineage subpopulation MHC II^lo^CD11b^+^Ly6C^hi^Ly6G^−^. Relative expression of Arg-1 was significantly increased in days 1 and 7 compared to control (relative expression of Arg-1; 1.62 ± 0.73 in day 1 vs. 2.21 ± 0.48 in day 7, **p* < 0.05, ****p* < 0.001). The Mann–Whitney *U* test was used to calculate the statistical significance. The mean values were quantified from independently repeated experiments three times.

Video S1 4D live imaging tracking of dorsally transplanted femur bone graft (scale bar = 30 µm).

Video S2 3D structural analysis for vascular connections between the donor bone marrow and recipient fascia layer.

Video S3 GFP-expressing monocytes and macrophages (CXCR1-GFP, left side movie) and RFP expressing cancer cells (MCF7-RFP, right side movie) in bone marrow environment (scale bar = 50 µm, time stamp: hh/mm/ss).

Video S4 4D tracking for an active cancer cell (Pan02-RFP) in the bone marrow environment (scale bar = 50 µm).

Video S5 Focused view of 4D tracking for Panc02-RFP cells in the bone marrow environment (scale bar = 25 µm).

Video S6 Active interaction between MCF7-RFP cells and CX3CR1-GFP positive cells in early phase of cancer cell entry into the bone marrow environment (scale bar = 50 µm).

Video S7 The bone marrow environment 1 h after gemcitabine injection intravenously (scale bar = 50 µm).

Video S8 The bone marrow environment 24 h after gemcitabine injection intravenously (scale bar = 50 µm).

Video S9 The bone marrow environment 144 h after gemcitabine injection intravenously (scale bar = 50 µm).

Video S10 Live cell imaging for monoculture of cancer cells (MCF7-RFP) during 36 h *in vitro* (time stamp: hh/mm/ss, scale bar = 100 µm).

Video S11 Live cell imaging for cancer cells (MCF7-RFP) coculture with NIH/3T3 during 36 h *in vitro* (time stamp: hh/mm/ss, scale bar = 100 µm).

Video S12 Live cell imaging for cancer cells (MCF7-RFP) coculture with mouse bone marrow cells during 36 h *in vitro* (time stamp: hh/mm/ss, scale bar = 100 µm).
