# Supplementary figures and images for: Real-Time Monitoring of Cancer Cells in Live Mouse Bone Marrow
Source: Front Immunol. 2018 Aug 2;9:1681. doi: 10.3389/fimmu.2018.01681 (PMC6082970; doi:10.3389/fimmu.2018.01681)

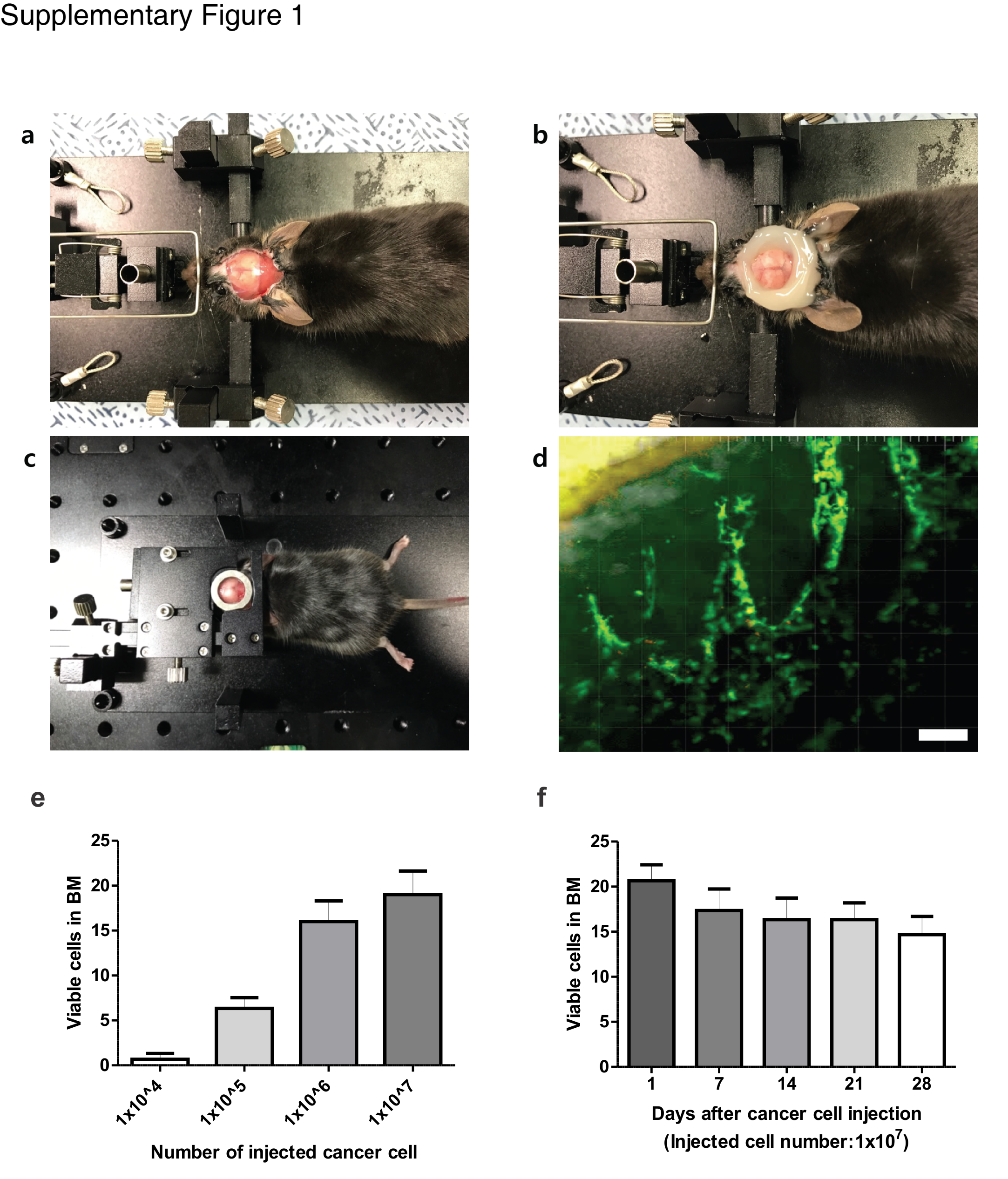

Supplement: Figure S1 — Calvarial bone marrow imaging model. (A) The scalp was removed by scissors with a 1.5-cm radius size. (B) Acrylic resin was applied around the exposed skull area to attach a fixation ring for the attachment with imaging mount module. (C) The fixation ring was attached to the pre-applied acrylic resin and then assembled with stereotactic head fixation device attached to a heating plate. (D) Two-photon microscopy revealed the typical bone marrow structure of the calvarium with trabecular anatomical structures in cortical bone (scale bar = 50 µm). (E) The number of viable cancer cells (MCF7) in calvarial bone marrow according to the number of injected cancer cells. (F) The number of viable cancer cells (MCF7) in calvarial bone marrow according to the period after cancer cell injection, from 1 to 28 days (the number of injected cancer cells: 1 × 10^7^). [file Image_1.jpeg]

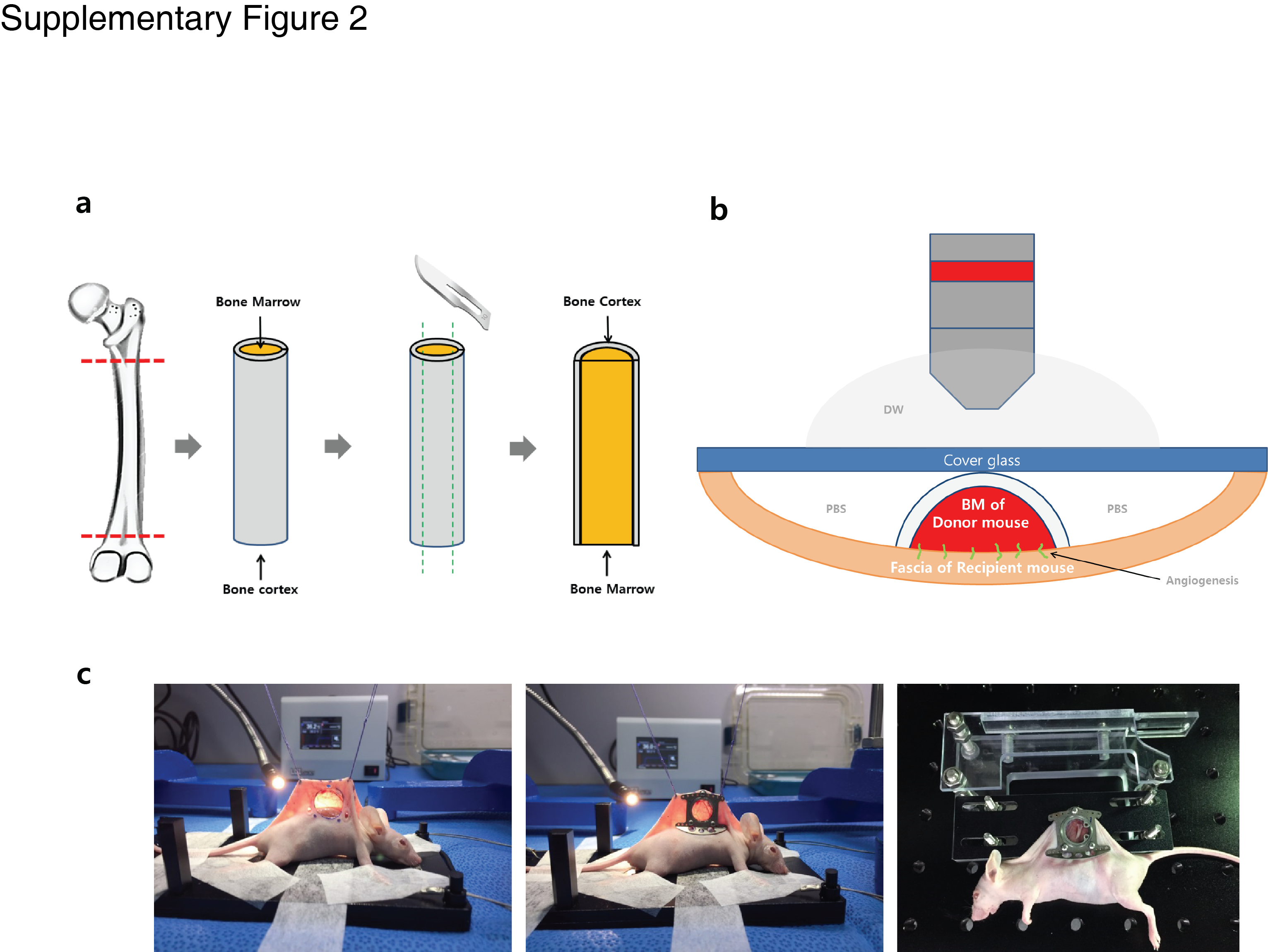

Supplement: Figure S2 — Dorsally transplanted femur bone marrow imaging model. (A) Bone graft processing from donor femur bone. (B) Final visualization of dorsal window for transplanted femur bone marrow using two-photon microscopy. (C) Operative procedures and mounting for intravital imaging of dorsal chamber implantation into recipient mouse. Detailed operative procedures are described in Section “Materials and Methods.” [file Image_2.jpeg]

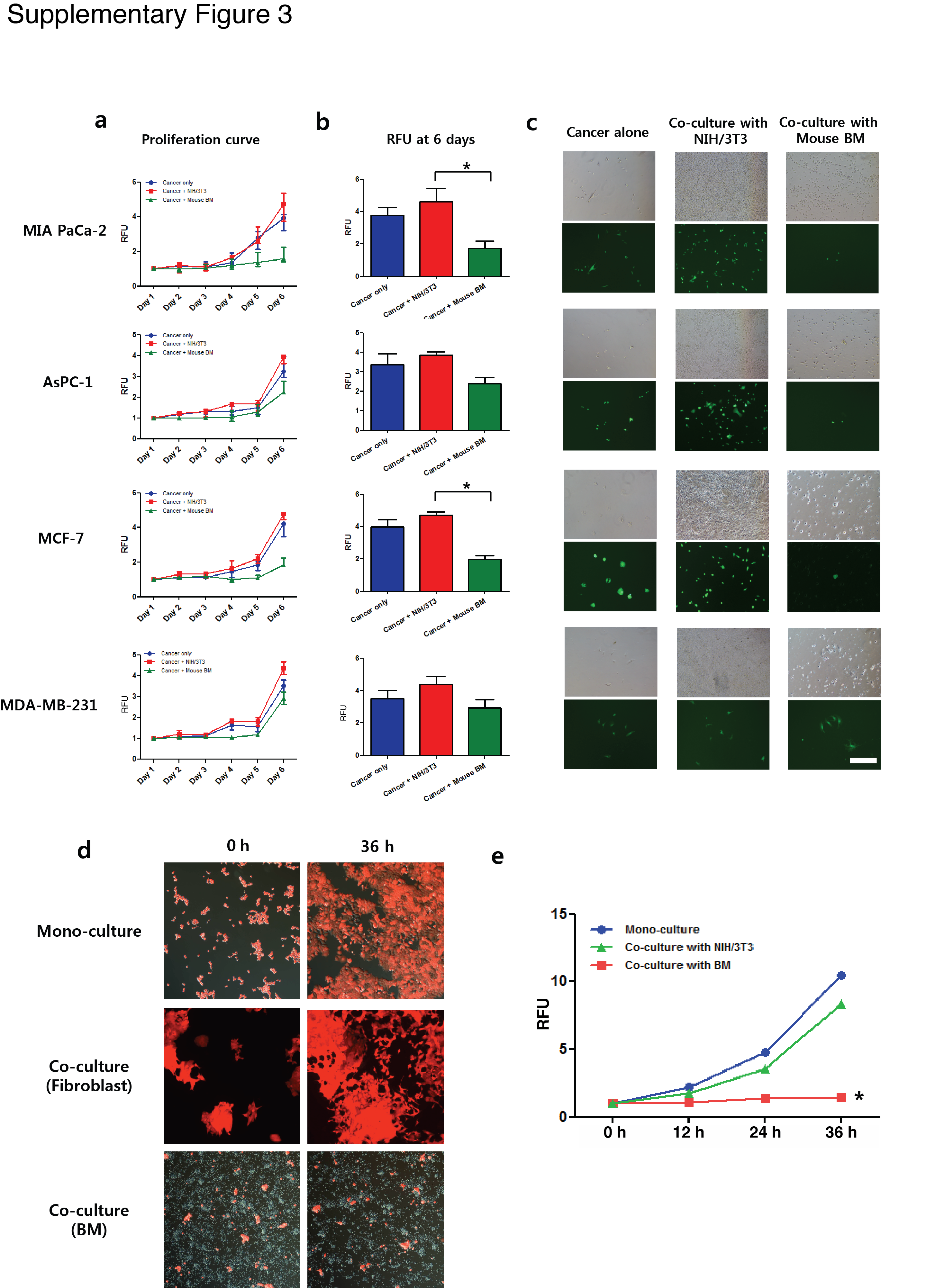

Supplement: Figure S3 — Human cancer cell lines adapt to bone marrow environment by dormant phenotype. (A) Proliferation assay for cancer cells with or without coculture to identify the inhibition effect of cancer cell proliferation by bone marrow stromal cells. Cancer cells (MIA PaCa-2, AsPC-1, MCF-7, and MDA-MB-231) alone or in coculture with NIH/3T3 (mouse fibroblast) cells or mouse bone marrow stromal cells that were aspirated from the femur bone of C57BL/6 mice. Coculture was performed on a 6-well plate (cancer vs. fibroblast or BM stromal cell = 1:10 ratio). (B) Relative fluorescence units on culture day 6. Proliferation of cancer cells coculture with mouse BM stromal cells compared to NIH/3T3 was significantly decreased in MIA PaCa-2 and MCF7 cell lines in the Mann–Whitney U test (relative fluorescence unit of MIA PaCa-2 in day 6 relative to day 1; 4.42 ± 1.18 in coculture with NIH/3T3 vs. 1.65 ± 0.52 in coculture with mouse BM stromal cells, MCF7; 4.47 ± 0.34 in coculture with NIH/3T3 vs. 1.74 ± 0.27 in coculture with mouse BM stromal cells, *p < 0.05). Data were averaged from independently repeated experiments three times. (C) Microscopy images of each cell line under bright field and green fluorescence. Bar graphs represent mean ± SD, scale bar = 30 µm. (D) Live cell imaging and (E) quantitative analysis of MCF7 cells showed restricted proliferation compared to active proliferation in monoculture and coculture with NIH/3T3 cells in the Mann–Whitney U test. *p < 0.05. See Videos S10–S12 in Supplementary Material. Representative data were shown from independently repeated experiments three times. [file Image_3.jpeg]

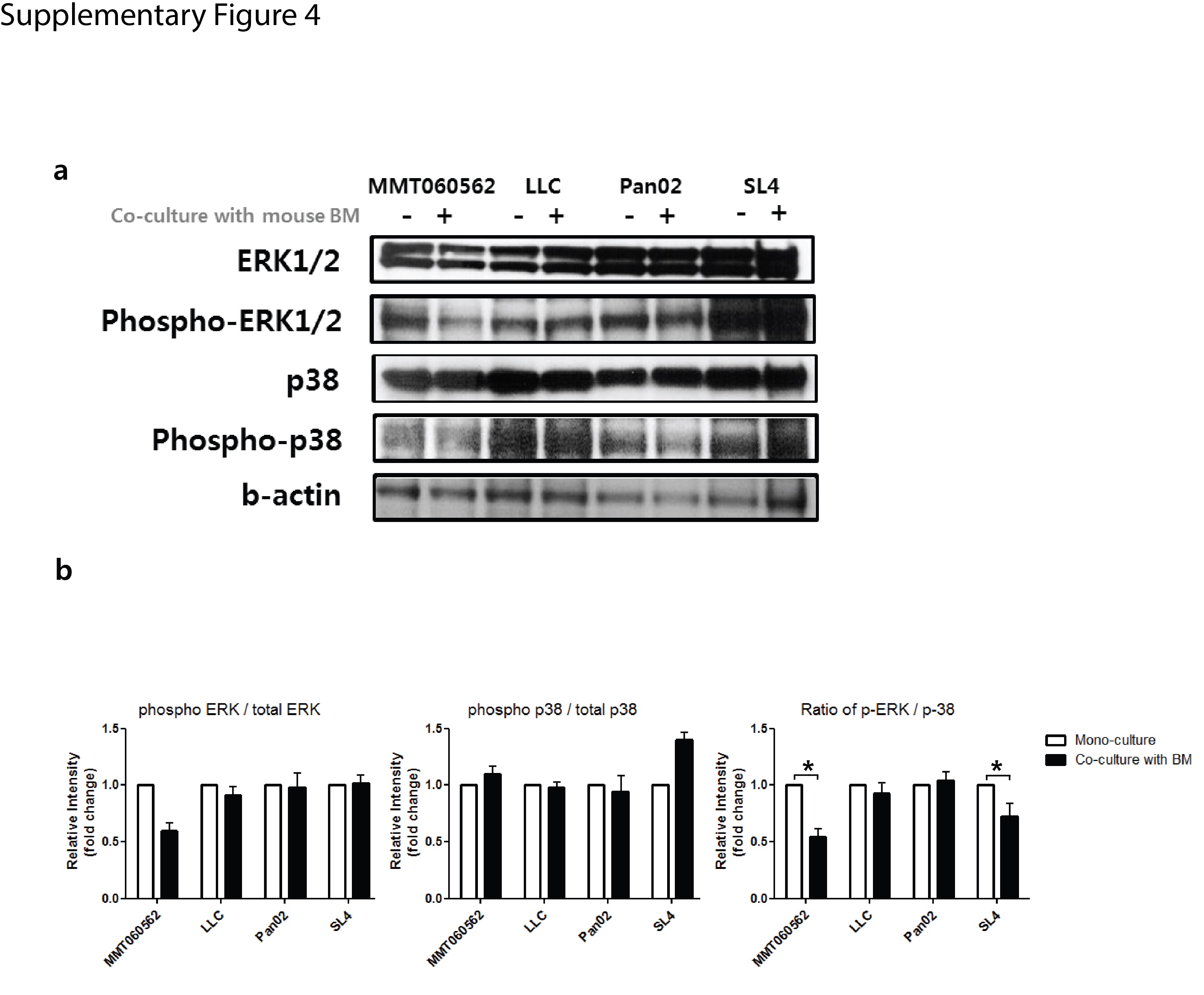

Supplement: Figure S4 — Western blot analysis of ERK/p-ERK/p38/p-p38 in mouse cancer cell line monocultures vs. coculture with mouse bone marrow. (A) Western blot to confirm of cancer dormancy at the molecular level. Various cancer cell-lines originated from a C57BL6 mouse (MMT060562, LLC, Pan02, SL4) were cocultured with mouse bone marrow stromal cells that were aspirated from the femur bone of a C57BL6 mouse. Blotting images represent the relative protein expressions of p-ERK/ERK and p-p38/p38 in cancer cell-lines. (B) Quantitative analysis of western blot images with or without coculture with mouse bone marrow by the Mann–Whitney U test. Expression ratio of phospho-ERK with phospho-p38 between monoculture and coculture with BM were significantly different in MMT060562 and SL4 cell lines. *p < 0.05. The mean values were quantified from pooled experiments conducted using different lysates from independent samples three times. [file Image_4.jpeg]

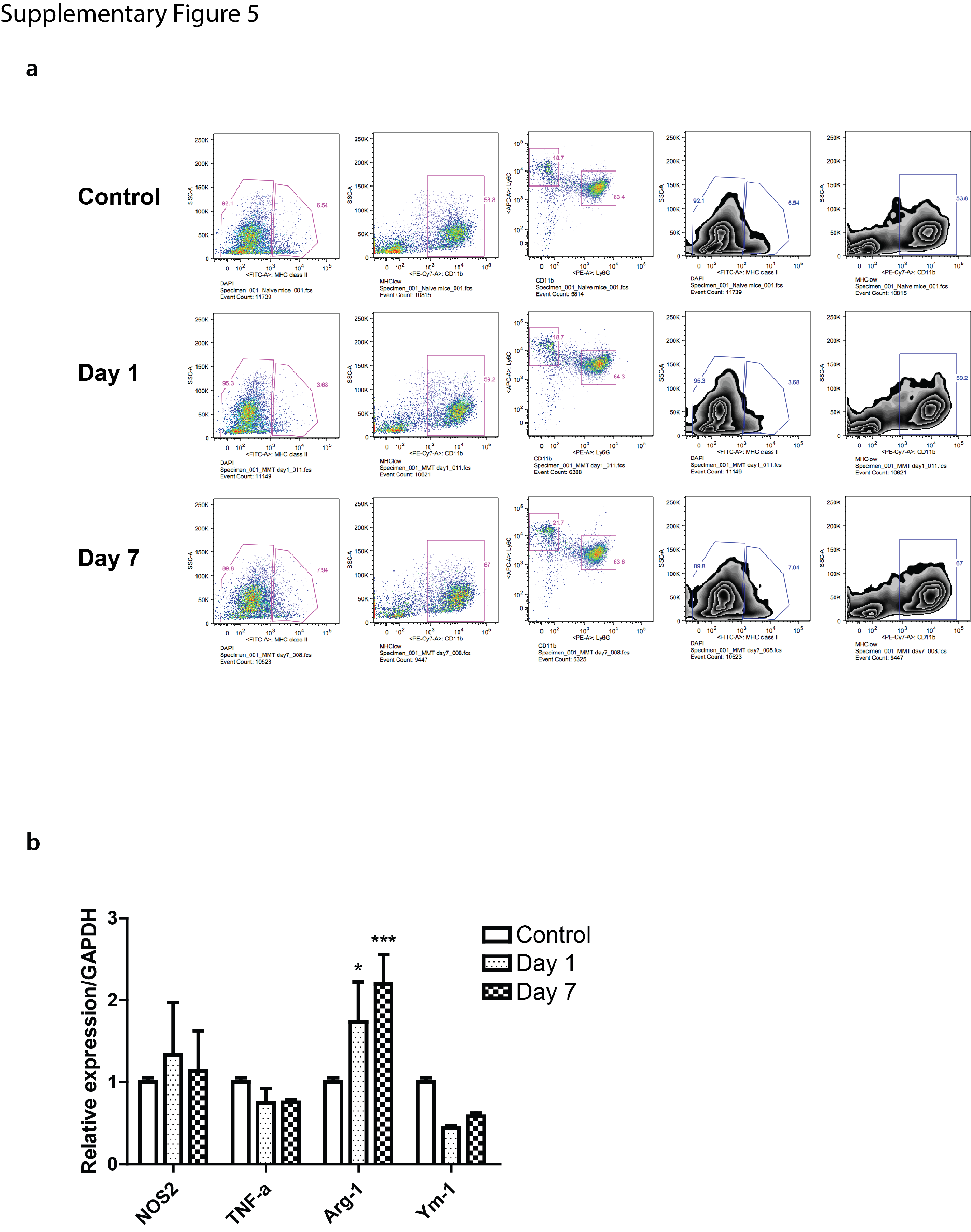

Supplement: Figure S5 — Temporal changes of myeloid lineage in mouse bone marrow after cancer cell injection via tail vein. (A) Chronological flow cytometry analysis showing control and 1 and 7 days after cancer cell injection via the tail vein. Injectable saline without cancer cells was injected to control mice. The acquisition of bone marrow was performed by the aspiration from bone marrow of the femur bone at the day of injection (control) and 1 and 7 days after injection via the tail vein. (B) Quantitative analysis of temporal changes for myeloid derived suppressive factors in myeloid lineage subpopulation MHC IIloCD11b+Ly6ChiLy6G−. Relative expression of Arg-1 was significantly increased in days 1 and 7 compared to control (relative expression of Arg-1; 1.62 ± 0.73 in day 1 vs. 2.21 ± 0.48 in day 7, *p < 0.05, ***p < 0.001). The Mann–Whitney U test was used to calculate the statistical significance. The mean values were quantified from independently repeated experiments three times. [file Image_5.jpeg]
